# Temporal patterns of sitting at work are associated with neck–shoulder pain in blue-collar workers: a cross-sectional analysis of accelerometer data in the DPHACTO study

**DOI:** 10.1007/s00420-016-1123-9

**Published:** 2016-03-02

**Authors:** David M. Hallman, Svend Erik Mathiassen, Marina Heiden, Nidhi Gupta, Marie Birk Jørgensen, Andreas Holtermann

**Affiliations:** 1 Department of Occupational and Public Health Sciences, Centre for Musculoskeletal Research, University of Gävle, Kungsbäcksvägen 47, 801 76 Gävle, Sweden; 2 National Research Centre for the Working Environment, Copenhagen, Denmark

**Keywords:** Neck pain, Sedentary, Time pattern, Physical activity, Occupational health

## Abstract

**Background:**

Our aim was to examine the extent to which temporal patterns of sitting during occupational work and during leisure-time, assessed using accelerometry, are associated with intense neck–shoulder pain (NSP) in blue-collar workers.

**Methods:**

The population consisted of 659 Danish blue-collar workers. Accelerometers were attached to the thigh, hip, trunk and upper dominant arm to measure sitting time and physical activity across four consecutive days. Temporal sitting patterns were expressed separately for work and leisure by the proportion of total time spent sitting in brief bursts (0–5 min), moderate (>5–20 min) and prolonged (>20 min) periods. The peak NSP intensity during the previous 3 months was assessed using a numerical rating scale (range 0–10) and dichotomized into a lower (≤4) and higher (>4) NSP score. Logistic regression analyses with multiple adjustments for individual and occupational factors were performed to determine the association between brief, moderate and prolonged sitting periods, and NSP intensity.

**Results:**

Time in brief bursts of occupational sitting was negatively associated with NSP intensity (adjusted OR 0.68, 95 % CI 0.48–0.98), while time in moderate periods of occupational sitting showed a positive association with NSP (adjusted OR 1.32, 95 % CI 1.04–1.69). Time in prolonged periods of occupational sitting was not associated with NSP (adjusted OR 0.78, 95 % CI 0.78–1.09). We found no significant association between brief, moderate or prolonged sitting periods during leisure, and NSP.

**Conclusion:**

Our findings indicate that the association between occupational sitting time and intense NSP among blue-collar workers is sensitive to the temporal pattern of sitting.

## Background

Excessive sitting has been proposed to be a determinant of upper extremity musculoskeletal disorders (MSDs) in the working population (Ariëns et al. 2001). Several cross-sectional studies have found a positive association between the duration of occupational sitting and occurrence of pain in the neck–shoulder region (Ariëns et al. 2000; Cagnie et al. 2007; Hallman et al. 2015b; Skov et al. 1996; Yue et al. 2012), while prospective studies on sitting and neck–shoulder pain, albeit few, are inconclusive (Ariëns et al. 2001; Mayer et al. 2012).

It is well documented that white-collar workers spend a substantial proportion of their time at work sitting (Ryan et al. 2011; Thorp et al. 2012; Toomingas et al. 2012). Thus, investigations of associations between sitting and neck–shoulder disorders are often conducted on workers in what is usually considered “sedentary” occupations (Cagnie et al. 2007; Skov et al. 1996), such as office-based jobs. However, recent studies based on objectively measured sitting time show that prolonged occupational sitting also occurs in blue-collar occupations such as manufacturing and construction (Gupta et al. 2015). These workers may even sit extensively during their leisure-time (Hallman et al. 2015a).

In a previous study on 202 blue-collar workers (Hallman et al. 2015b), we found that sitting, measured using accelerometry, for more than a total of 8.2 h a day was associated with increased pain intensity from the neck–shoulder region, compared to moderate sitting, in the range from 6.5 to 8.2 h. We also found that, among males, sitting little (<2.0 h) at work was associated with reduced pain intensity, compared to moderate sitting (i.e., 3.7–6.6 h), even after adjustment for several other occupational risk factors. While these results suggest that sitting may show an association with neck–shoulder pain in blue-collar work regardless of established risk factors, including heavy lifting and awkward postures (Côté et al. 2009; Palmer and Smedley 2007), it is still not clear whether extensive sitting is associated with pain in its own right, or just a proxy for other important risk factors. In order to disentangle this question, associations between sitting and pain need to be examined in more detail, accounting, for instance, for important biomechanical exposures that may be correlated with sitting, such as constrained upper extremity postures or low levels of physical activity during work and leisure (Ariëns et al. 2001; Hildebrandt et al. 2000; Mayer et al. 2012).

Epidemiological and experimental studies suggest that the temporal pattern of sitting (or “sedentary behavior”) is an important determinant of essential health outcomes (Carson et al. 2014; Healy et al. 2008; Henson et al. 2013), including MSDs (Thorp et al. 2014). Breaking up prolonged sitting by periods of standing or walking has shown beneficial effects compared to uninterrupted sitting on the regulation of cardiovascular (Larsen et al. 2014; Thosar et al. 2014) and pro-inflammatory biomarkers (Henson et al. 2013; Latouche et al. 2013; Yates et al. 2012) suggested to be involved in causal pathways of neck–shoulder pain (Barbe and Barr 2006; Bruehl and Chung 2004). This agrees well with the more general notion in occupational health research and practice that variation in biomechanical exposure is important for musculoskeletal health and well-being (Mathiassen 2006; Straker and Mathiassen 2009). It therefore appears reasonable to expect that a possible relationship between sitting and neck–shoulder pain would depend on the temporal pattern of sitting, including whether it is accumulated in periods of longer or shorter durations. Specifically, sitting in long uninterrupted periods could be expected to show a positive association with neck–shoulder pain, while the opposite relationship would occur for short periods in sitting.

A thorough analysis of temporal sitting patterns needs to be based on objective measurement data, as self-reported measures of sitting cannot be expected to operate at the time resolution required for a detailed record of sitting and non-sitting periods, and furthermore are prone to bias and insufficient precision (Celis-Morales et al. 2012; Clark et al. 2011). The common use of self-reports may be one important reason that studies reporting temporal sitting patterns in detail are rare (Thorp et al. 2012; Toomingas et al. 2012), particularly among blue-collar workers.

Our aim was to investigate the extent to which temporal patterns of occupational and leisure-time sitting, as assessed using accelerometry, are associated with intense neck–shoulder pain among blue-collar workers. We hypothesize that the proportion of time spent in moderate and prolonged, uninterrupted periods of sitting is positively associated with intense pain, while the opposite association holds for the occurrence of short sitting periods.

## Methods

### Study design and population

The present study is a part of the Danish PHysical ACTivity cohort with Objective measurements (DPHACTO). The main objective of DPHACTO is to investigate the association between objectively measured physical activities at work and frequent prospective measurements of musculoskeletal pain among blue-collar workers. The complete study protocol is described in detail elsewhere (Jørgensen et al. 2013).

**Fig. 1:**
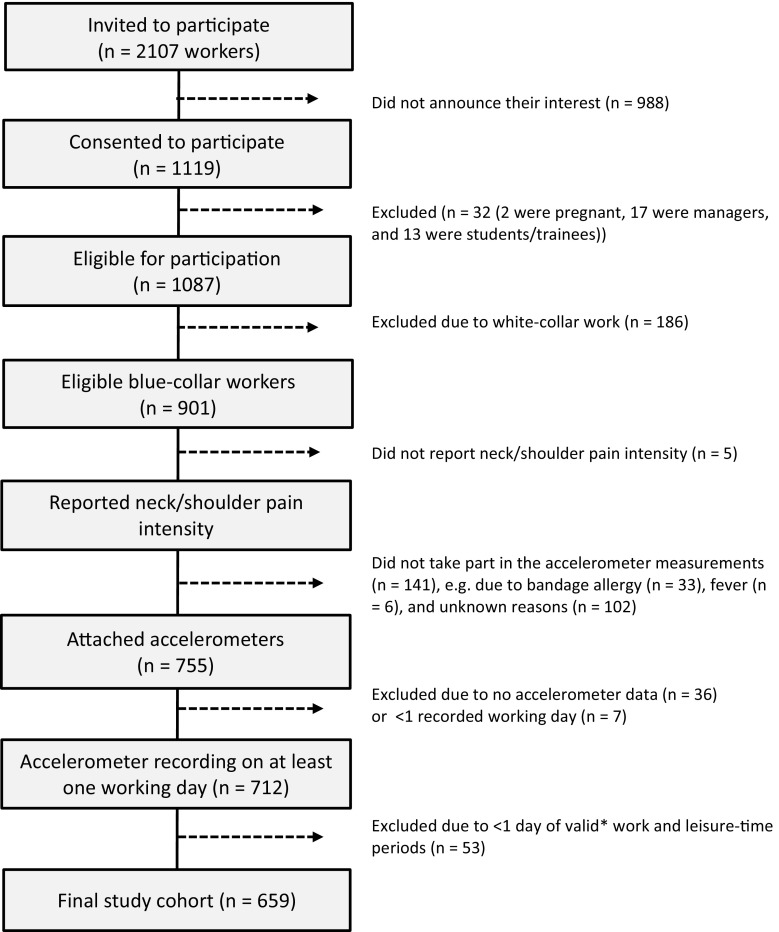
Recruitment of participants. *Valid (work and leisure) is defined as at least 4 h or 75 % of the average duration of work and leisure for a particular worker

The present study is a cross-sectional analysis of data from the baseline measurements. Data were collected from spring 2012 to spring 2013 at workplaces within three different occupational sectors (i.e., cleaning, transport and manufacturing) in Denmark. Employees (*n* = 2107) from 15 companies were invited to participate (see Fig. 1 for the recruitment of participants). Workplaces were considered eligible if they allowed measurements to be collected during working hours. Participants were included if they reported blue-collar work as their main occupation. Exclusion criteria were predominant white-collar work, managing position, pregnancy and allergy to adhesives.

Eligible blue-collar workers (*n* = 901) were invited to fill in a short baseline questionnaire, to undergo a health check and a physical examination, and to take part in field measurements, including objective exposure data collection across four consecutive days. Data on self-reported neck–shoulder pain were obtained from 896 workers, among whom 712 were subjected to accelerometer measurements, resulting in valid measures from 659 workers.

All workers provided their written informed consent prior to participation. The present study was conducted according to the Helsinki Declaration and approved by the Danish data protection agency and local ethics committee (H-2-2012-011).

### Procedure

The participants were asked to wear four accelerometers (see below) around the clock during four consecutive days, including at least two working days. The participants were instructed to wear the equipment the whole measurement period, and to perform a reference measurement in upright stance for 15 s each day, to secure accurate activity detection from the accelerometer signals. They were also instructed to remove the equipment if it caused any kind of discomfort. During the measurement period, a paper diary was used by the participant to note working hours, leisure-time, and time for going to bed in the evening and waking up in the morning, as well as time of the reference measurements. At the end of the four-day data collection, the equipment was returned to the research staff.

### Accelerometry

The participants were equipped with triaxial accelerometers (Actigraph GT3X+, ActiGraph LLC, Florida, USA) placed on the thigh, dominant upper arm, hip and trunk, using previously described procedures (Gupta et al. 2015; Hallman et al. 2015b; Skotte et al. 2014). Acceleration data were sampled at a frequency of 30 Hz with a dynamic range of ±6G and a 12-bit precision. The accelerometers were initialized for recording and downloading of data using the Actilife software version 5.5 (ActiGraph LLC, Pensacola, FL, USA), while the data obtained from the accelerometers were processed off-line and analyzed using a custom-made MATLAB-based software, Acti4 (The National Research Centre for the Working Environment, Copenhagen, Denmark and BAuA, Berlin, Germany), which determines the type and duration of different activities and body postures with a high sensitivity and specificity, both in controlled experiments and free-living conditions (Ingebrigtsen et al. 2013; Korshøj et al. 2014; Skotte et al. 2014; Stemland et al. 2015).

Non-wear was judged to occur when (a) the software detected a period longer than 90 min with zero acceleration counts, or (b) the participant reported non-wear-time, or (c) artefacts or missing data were detected by visual inspection. Non-work days, bedtime and sleep-periods were also excluded from further analyses. Each work and leisure-time interval had to contain at least 4 h/day of accelerometer wear-time or 75 % of the average wear-time across days for the individual. The overall accelerometer non-wear-time in the final study population was 0.5 % for the thigh and 0.5 % for the hip/trunk accelerometers.

### Assessment of sitting time

The occurrence of sitting periods was identified from the accelerometer outputs based on previously described procedures (Gupta et al. 2015; Skotte et al. 2014; Stemland et al. 2015). Sitting was detected using the signals from the thigh and trunk accelerometers, while data from the hip accelerometer (if available) were used for periods classified as non-wear-time for the trunk accelerometer. In brief, the accelerometer signals were first low-pass filtered at 5 Hz using a fourth-order Butterworth filter and then split up in 2-s windows with 50 % overlap. Sitting periods were then determined to occur when thigh inclination was above 45° *and* trunk inclination was below 45° relative to the recorded reference position, i.e., upright standing (Gupta et al. 2015). The temporal sitting pattern was quantified using exposure variation analysis, EVA (Mathiassen and Winkel 1991). Based on the time line of the processed accelerometer signal for each measurement day, the occurrence of uninterrupted sitting periods of different durations were derived from work and leisure-time, respectively. Interruptions from sitting were required to be at least 5 s to qualify as a non-sitting period. Three EVA derivatives were selected based on Ryan et al. (2011) and Straker et al. (2014): “brief bursts” (time in sitting periods ≤5 min), “moderate periods” (time in sitting periods of >5–20 min) and “prolonged periods” (time in sitting periods >20 min). For each worker, the mean time (h/day) spent during work and during leisure in each of these categories (i.e., ≤5 min, >5–20 min, >20 min) was calculated by dividing the total accumulated sitting time in that category across all measurement days by the number of days. Then, these values were expressed in percent of the daily average of total wear-time at work and leisure, respectively.

### Assessment of neck–shoulder pain intensity

Self-reported information about neck and shoulder pain intensity was obtained using the Standardized Nordic Questionnaire for the analysis of musculoskeletal symptoms (Kuorinka et al. 1987). Peak pain intensity in the neck–shoulder region during the previous 3 months was rated on a numeric rating scale (NRS), ranging from 0 (“no pain”) to 10 (“worst pain imaginable”). The NRS is a valid instrument for assessment of pain intensity (Ferreira-Valente et al. 2011), and it has been recommended as a “core outcome measure” by the “Initiative on Methods, Measurement, and Pain Assessment in Clinical Trials,” IMMPACT (Dworkin et al. 2005). As the pain intensity scores were not normally distributed, scores were categorized into “low” (0–4) and “high” (>4) pain intensities prior to further analysis. This cut-point has previously been shown to have clinical relevance (Andersen et al. 2012). Also, for descriptive purposes, the number of days with pain was assessed using the question “In the past 12 months, how many days in all have you had pain or discomfort in the neck/shoulders?” with six response categories ranging from “0 days” to “every day.”

### Assessment of possible confounders

A large selection of individual and occupational factors were chosen a priori as potential confounders or effect modifiers based on previous literature and theoretical assumptions concerning their possible influence on sitting behavior and neck–shoulder pain.

Age was determined from the workers’ Danish civil registration numbers, while smoking was assessed by the question “Do you smoke?” using four response categories, which were merged into a dichotomized variable: yes (“yes daily”, “yes sometimes”) and no (“used to smoke”, “I have never smoked”). Body mass index (BMI, kg m^−2^) was calculated from objectively measured height (cm) and body weight (kg). Seniority in the current job (months) was assessed using the question: “For how long have you had the kind of occupation that you have now?” Lifting and carrying at work was assessed using a single item from the Danish Work Environment Cohort Survey (DWECS): *How much of your working time do you carry or lift?*, using a six-point response scale ranging from 1 (“never”) to 6 (“almost all the time”) (Tüchsen et al. 2006). Psychosocial factors at work were assessed using four items from the Copenhagen Psychosocial Questionnaire (Pejtersen et al. 2010) representing two dimensions, i.e., influence at work (decision authority): “Do you have a large degree of influence concerning your work?”; “Can you influence the amount of work assigned to you?” and Social support: “Is there good co-operation between the management and the employees?”; “Is there good co-operation between the colleagues at work?” The five-point response scale ranged from 1 (“always”) to 5 (“never”). After reversing the scale and recoding it to 0–4, answers to the two items were added up to a 0–8 scale for each dimension according to the questionnaire manual (available at: www.arbejdsmiljoforskning.dk), whereby higher numbers indicate more influence and better social support, respectively.

Physical activity was assessed using data from the accelerometers described above (Ingebrigtsen et al. 2013; Stemland et al. 2015). The total time (h/day) spent in walking, climbing stairs, running and cycling was added up separately for work and leisure. Sitting (h/day) with the dominant upper arm elevated >90° was estimated from the accelerometer signals according to Korshøj et al. (2014) for work and leisure separately.

### Statistical analyses

All statistical analyses were performed in IBM SPSS Statistics 22.0 for Windows. Binary logistic regression analyses were performed to determine the association between temporal sitting patterns and intense neck–shoulder pain. The regression models were performed in two steps (crude and adjusted models), using the dichotomized peak pain intensity variable (low pain 0–4; intense pain 5–10) as an outcome. First, the independent variables consisted of the EVA derivatives only, i.e., time (%) spent sitting in brief, moderate and prolonged periods, which were entered together in the same model (crude model). Second, in addition to the EVA derivatives, the potential confounders (described above), except for psychosocial factors, as well as interaction terms between gender and each EVA derivative were included (adjusted model). Due to the skewed distribution of the EVA derivatives and the covariate “Sitting with upper arm elevated >90°,” these variables were square root (*sqrt*)-transformed prior to the analyses, which resulted in closer to normal distributions. Each analysis was performed for work and leisure-time separately.

To determine whether the results were consistent when also accounting for psychosocial factors, the adjusted models were refitted using self-reported influence and social support at work as additional covariates. These two covariates were not included in the first adjusted analysis because they caused a reduction of the sample size, i.e., from *n* = 659 to *n* = 458, due to missing values.

In order to determine whether the association between the temporal pattern of occupational sitting and pain intensity was consistent across different levels of total occupational sitting time, additional logistic regression analyses were performed on data stratified on total sitting time at work (more/<25 % of total work time spent sitting) and on total sitting time in leisure (more/<50 % time spent sitting), both cut-points being close to the median values in the population. Finally, all regression analyses were also performed on EVA derivatives in absolute time (*sqrt* h/day) rather than proportion of time, as used above.

Data are presented in text and tables as means with standard deviations between subjects, or frequencies and proportions, if not otherwise stated. For each regression model, odds ratios (OR) and 95 % confidence intervals (CI) were derived. Associations with *p* values <.05 were considered significant.

## Results

**Fig. 2:**
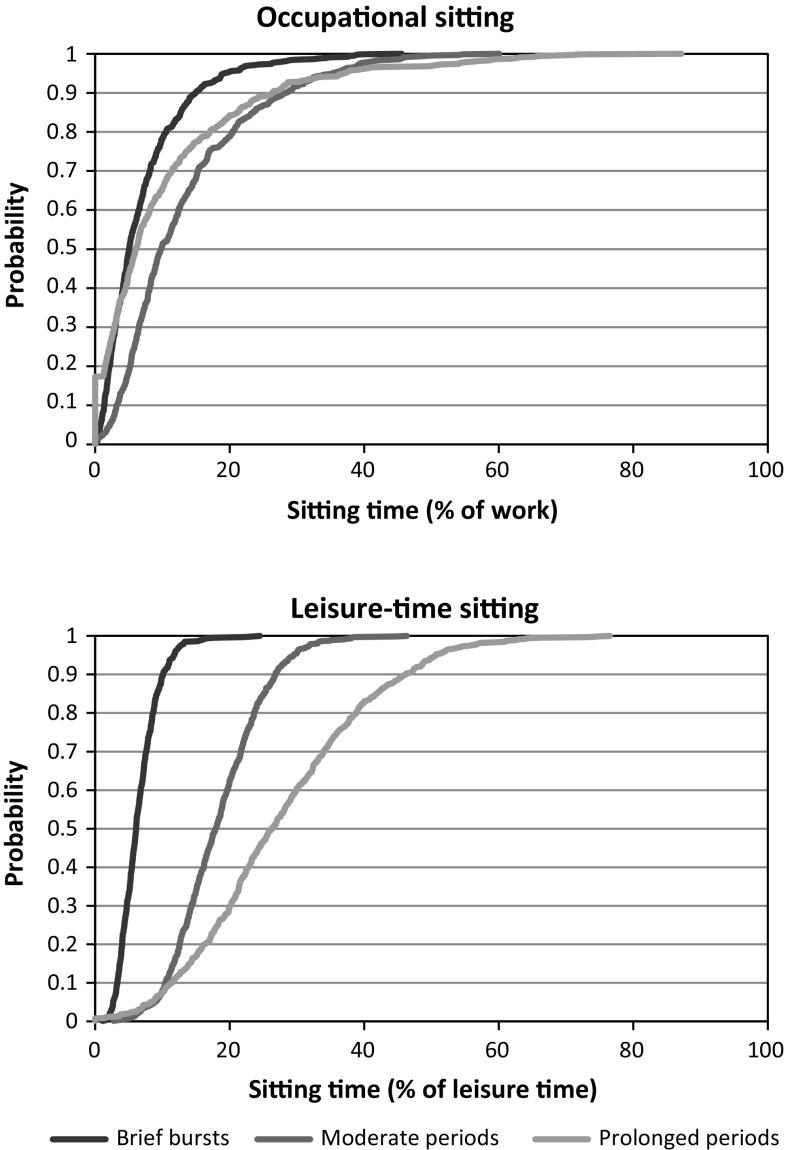
Cumulative probability distributions of EVA derivatives in the study population, i.e., *brief bursts* (time in sitting periods ≤5 min), *moderate periods* (time in sitting periods of >5–20 min) and *prolonged periods* (time in sitting periods >20 min) for occupational and leisure-time sitting, respectively

Objective measurements of sitting time were collected from 659 blue-collar workers, including males (*n* = 363) and females (*n* = 296) from three occupational sectors, i.e., cleaning, manufacturing, and transportation (Table 1). The age of the workers ranged between 18 and 68 years, and they had been in their current job for, on average, 13 years (SD 10). About 31 % of the workers were smokers. Accelerometer data were collected for, on average, 2.6 (SD 1.0) days per worker, comprising 19.9 (SD 8.0) and 23.0 (SD 9.1) h per worker of valid recordings during work and leisure-time, respectively. Cumulative distributions of uninterrupted sitting time in brief, moderate and prolonged periods are shown in Fig. 2.

**Table 1:** Descriptive data on 659 blue-collar workers with accelerometer measurements of sitting time

	*n*	*n* (%)	Mean	SD
Age (years)	659		45.0	9.9
Gender	659			
Females [*n* (%)]		296 (44.9)		
Smokers [*n* (%)]	641	196 (30.6)		
Sector				
Cleaning [*n* (%)]		128 (19.4)		
Manufacturing [*n* (%)]		470 (71.3)		
Transportation [*n* (%)]		61 (9.3)		
Body mass index (kg/m^2^)	649		27.5	4.9
Seniority (years)	635		13.0	10.2
Influence at work (scale 0–8)	458		4.9	2.1
Social support at work (scale 0–8)	458		6.3	1.3
Lifting and carrying time at work (scale 1–6)	661		3.5	1.4
Valid work per day (h/day)	659		7.59	1.28
Valid leisure per day (h/day)	659		8.84	1.69
Total valid work (h)	659		19.86	8.05
Total valid leisure (h)	659		23.01	9.11
Occupational sitting (% work time)	659		30.1	20.2
Leisure-time sitting (% leisure-time)	659		52.0	12.5
Sitting at work with upper arm above 90° (h/day)	643		0.02	0.03
Sitting at leisure with upper arm above 90° (h/day)	643		0.12	0.18
Physical activity at work (h/day)	659		1.29	0.55
Physical activity during leisure (h/day)	659		0.86	0.40
Peak neck–shoulder pain intensity (scale 0–10)	659		3.4	3.0
Pain intensity ≤4 [*n* (%)]		413 (62.7)		
Pain intensity >4 [*n* (%)]		246 (37.3)		
Days with neck/shoulder pain previous year	659			
0 days [*n* (%)]		172 (26.1)		
1–7 days [*n* (%)]		186 (28.2)		
8–30 days [n (%)]		134 (20.3)		
31–90 days [*n* (%)]		60 (9.1)		
>90 days [*n* (%)]		39 (5.9)		
Every day [*n* (%)]		68 (10.3)

Among the 659 workers, the peak pain intensity was, on average, 3.4 (SD 3.0) on a 0–10 scale. Thirty-seven percent of the workers reported a peak pain intensity score >4, and 63 % reported a pain intensity ≤4. Twenty-six percent of the workers reported 0 days with neck–shoulder pain over the past year, 48 % reported 1–30 days, and 25 % reported >30 days with pain.

### Primary analyses of the association between sitting patterns and neck–shoulder pain

**Fig. 3:**
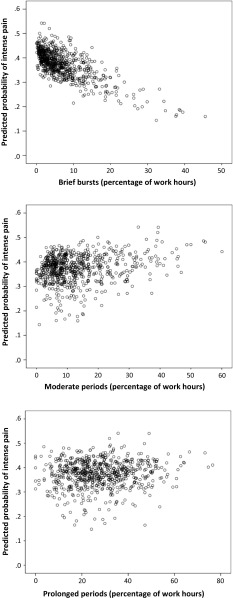
Crude association between temporal sitting patterns [*brief burst* (≤5 min), *moderate periods* (>5–20 min), *prolonged periods* (>20 min)] at work and neck–shoulder pain intensity. *X-axes* show the proportion of sitting time at work occurring in the two categories; *y-axes* show the predicted probability of reporting intense (>4, scale 0–10) neck–shoulder pain

The results from the crude and adjusted logistic regression models for occupational and leisure-time sitting patterns are shown in Table 2. We found that the temporal sitting pattern at work, expressed by EVA derivatives, was associated with the intensity of neck–shoulder pain. Specifically, we found a significant (*p* < .05) negative association between “brief bursts” (<5 min) of occupational sitting and pain intensity, and a positive association between “moderate periods” (>5–20 min) at work and pain intensity (Fig. 3). These associations remained significant after adjusting for multiple covariates, including several individual and biomechanical factors. We found no association between “prolonged periods” and pain intensity. We did not find any significant association between sitting patterns during leisure-time and neck–shoulder pain intensity. There was no significant main effect of gender, and no interaction between gender and the sitting variables in any of the models (all *p* > .05).

**Table 2:** Associations between temporal patterns (EVA derivatives) of occupational and leisure-time sitting and intense neck–shoulder pain (>4 on a 0–10 scale)

	*n*	B	*p*	OR	Lower 95 % CI	Upper 95 % CI
Occupational sitting patterns						
Crude model						
Brief bursts	659	−**0.27**	**.00**	**0.77**	**0.64**	**0.92**
Moderate periods		**0.16**	**.03**	**1.17**	**1.02**	**1.35**
Prolonged periods		−0.01	.85	0.99	0.91	1.08
Adjusted model^a^						
Brief bursts	595	−**0.38**	**.04**	**0.68**	**0.48**	**0.98**
Moderate periods		**0.28**	**.02**	**1.32**	**1.04**	**1.69**
Prolonged periods		−0.08	.33	0.92	0.78	1.09
Leisure-time sitting patterns						
Crude model						
Brief bursts	659	0.19	.25	1.21	0.87	1.69
Moderate periods		−0.04	.69	0.96	0.77	1.19
Prolonged periods		−0.01	.85	0.99	0.86	1.13
Adjusted model^a^						
Brief bursts	595	0.23	.44	1.25	0.71	2.21
Moderate periods		−0.27	.15	0.76	0.52	1.10
Prolonged periods		−0.11	.37	0.90	0.71	1.14

Odds ratios (ORs) indicate the relative increase in risk for reporting intense pain with each unit (*sqrt* percent time) increment in sitting

All sitting variables were normalized to percentages of total wear-time at work or leisure, and square-root-transformed prior to the logistic regression analyses. Significant (*p* < .05) associations are bold-faced

^a^Adjusted for age, gender, smoking, BMI, job seniority, lifting/carrying time at work, physical activity at work, physical activity during leisure, sitting with arms above 90° (either at work or at leisure depending on the modeled domain)

### Adjustment for psychosocial factors

Adding the psychosocial factors *influence at work* and *social support at work* as additional covariates in the primary (adjusted) logistic regression models did not change the results for occupational sitting to any notable extent (adjusted model: “brief bursts” OR 0.60, 95 % CI 0.40–0.91; “moderate periods” OR 1.23, 95 % CI 0.93–1.63; “prolonged periods” OR 0.84, 95 % CI 0.69–1.02), although the 95 % CIs became wider and now included 1.00 for *moderate periods*. All associations remained nonsignificant for leisure-time sitting.

### Stratification on total sitting time

The same logistic regression models as above were resolved with stratification for total sitting time at work (cut-point sitting more or less than 25 % of the working time). We found that the association with neck–shoulder pain intensity persisted for “brief bursts” (adjusted OR 0.49, 95 % CI 0.26–0.91) and “moderate periods” (adjusted OR 1.52, 95 % CI 0.94–2.45) in the lower total sitting time stratum (*n* = 332), with comparable ORs to those found in the primary models. The same trends, although nonsignificant, were observed in the higher sitting stratum (*n* = 327) for “brief bursts” (adjusted OR 0.62, 95 % CI 0.35–1.09) and “moderate periods” (adjusted OR 1.17, 95 % CI 0.81–1.70). *Prolonged periods* was not associated with pain in the lower (adjusted OR 0.98, 95 % CI 0.71–1.36) or higher sitting time strata (adjusted OR 0.80, 95 % CI 0.59–1.09). When stratifying for total leisure-time sitting (cut-point: sitting for 50 % of the leisure-time), all associations between EVA derivatives and neck pain remained nonsignificant in both the crude and adjusted models.

### Absolute sitting time instead of percentages

All the regression models were also carried out based on absolute sitting time (*sqrt* h/day) instead of percentages. For the pattern of occupational sitting, absolute values led to slightly stronger ORs for “brief bursts” (adjusted OR 0.27, 95 % CI 0.07–1.00) and “moderate periods” (adjusted OR 2.73, 95 % CI 1.15–6.52) than time proportions, while “*prolonged periods*” were still not significantly associated with pain (adjusted OR 0.74, 95 % CI 0.40–1.37). Associations with leisure-time sitting were similar for EVA metrics expressed in absolute values and percentages.

## Discussion

Our main findings were that occupational sitting spent in uninterrupted periods of brief and moderate duration showed opposite associations with intense neck–shoulder pain among blue-collar workers, even after adjusting for a range of individual and occupational factors of relevance to musculoskeletal pain. No significant association with pain was found for prolonged periods of occupational sitting, and temporal sitting patterns during leisure-time were not found to be associated with neck–shoulder pain at all. Thus, our results suggest that information on total sitting time only is not sufficient to appreciate the association between sitting exposure at work and neck–shoulder pain.

The current findings corroborate previous research suggesting a positive association between occupational sitting time and neck–shoulder pain (Ariëns et al. 2001; Cagnie et al. 2007; Hallman et al. 2015b; Skov et al. 1996; Yue et al. 2012), although negative results have also been reported (Holm et al. 2013), with one study reporting sitting time (>75 % of the working time) even to be associated with a favorable prognosis of neck–shoulder pain (Grooten et al. 2007). A possible reason for these inconsistent findings is that most previous studies have assessed exposure to sitting using self-reports (Celis-Morales et al. 2012; Clark et al. 2011). A major strength of the current study is the use of multiple triaxial accelerometers to objectively assess the uninterrupted time line of sitting and non-sitting across several days. This also allowed us to derive detailed temporal sitting patterns with high accuracy, which would not have been possible using questionnaires. By combining data from three accelerometers, we could discriminate sitting from lying and standing, while also monitoring arm movements during sitting, as well as walking, running and cycling, which are believed to be important exposures for work-related pain. Also, our study population is very large compared to previous studies using direct measurements of occupational biomechanical exposures, including sitting, and the recruited blue-collar worker are rather homogenous with respect to socioeconomic status, which will minimize socioeconomic confounding. Exposure variation analysis (EVA) approach is a generic tool for retrieving important elements of the temporal structure (i.e., the variation) in physical exposure, and it has been used in previous accelerometer-based studies for analyzing time patterns of physical activity and sedentary behavior (Hallman et al. 2015a; Straker et al. 2014). Still, very few studies have so far investigated the utility of EVA when disentangling associations between temporal patterns of exposure, and health outcomes. The results of the present study suggest that the metrics produced by the EVA method are clinically relevant.

Our findings are in line with the stated hypothesis that time spent in short sitting periods at work would be negatively associated with intense neck–shoulder pain. Specifically, we found that the likelihood of reporting a pain intensity score >4 (i.e., “intense” pain) was reduced with increasing occurrence of occupational sitting in uninterrupted periods shorter than 5 min. Also, we found the inverse relationship for moderate sitting periods at work: the likelihood of reporting intense neck–shoulder pain increased with increasing occurrence of sitting in uninterrupted periods lasting between 5 and 20 min (Fig. 3). However, in contrast to our hypothesis, we did not find any significant association between the occurrence of prolonged periods (>20 min) of occupational sitting and pain intensity. This may be explained by limited time accumulated in sitting periods exceeding 20 min during work for many of the workers (Fig. 2). Still, we consider the occurrence of sitting in our sample of blue-collar workers, as well as the dispersion among workers of temporal sitting patterns to be adequate for investigating associations with health outcomes both during work and leisure (Fig. 2).

Our finding of opposite associations for *brief bursts* and *moderate periods* of occupational sitting with neck–shoulder pain is in line with recent studies indicating that breaking up seated work with periods of standing or walking is associated with beneficial outcomes related to health (Carson et al. 2014; Healy et al. 2008; Henson et al. 2013), including, muscle fatigue and discomfort (Thorp et al. 2014). More time spent in brief sitting periods at work may be a sign of more variation in biomechanical loading of the musculoskeletal system, which may protect against MSDs (Mathiassen 2006; Toomingas et al. 2012). In contrast, “constrained” working postures maintained for long periods of time is an accepted occupational risk factor for neck–shoulder pain (Côté et al. 2009; Mayer et al. 2012). During uninterrupted sitting periods, work may be characterized by little posture variation and sustained low-intensity muscle contractions, which are considered potential causal determinants for work-related muscle pain (Visser and van Dieën 2006). In contrast, work performed in short sitting periods may less likely be associated with constrained upper extremity postures and sustained muscle activity, since it will probably be more dynamic even in these respects. Further, excessive sitting, as well as sitting in uninterrupted periods, is associated with changes in cardiovascular (e.g., increased blood pressure) and inflammatory markers (e.g., increased systemic levels of pro-inflammatory cytokines) (Henson et al. 2013; Larsen et al. 2014; Yates et al. 2012), which may, in turn, play important roles in mediating muscle pain via different peripheral and central mechanisms (Barbe and Barr 2006; Bruehl and Chung 2004). However, these theories on possible pathways explaining the relationship between sitting patterns and intense neck–shoulder pain remain to be verified.

The cross-sectional design of the current study precludes us from making inferences about the causal relationship between temporal sitting patterns and pain intensity. However, it is worth noting that previous studies did not find sitting time to be distributed differently across the day between workers with and without chronic neck–shoulder pain (Hallman et al. 2014; Hallman and Lyskov 2012). This suggests that the temporal pattern of occupational sitting has an influence on neck–shoulder pain intensity rather than the reversed causation.

In order to account for possible confounders or effect modifiers, we adjusted the statistical models for several individual and biomechanical risk factors of relevance to neck–shoulder pain and found persistent associations between occupational sitting and neck–shoulder pain. Also, to examine whether the associations determined in the crude and adjusted models persisted when accounting for the total time in occupational sitting, we stratified our population by total sitting time at work using a cut-point close to median sitting time, i.e., sitting for more or less than 25 % of the working hours. In both sitting time strata we found the associations between *brief bursts* and *moderate periods* in sitting, and neck–shoulder pain to be equally strong (ORs of similar sizes) as those obtained in the primary analysis, while not reaching statistical significance (Table 2). Thus, our results suggest that the temporal pattern of sitting at work is associated with neck–shoulder pain independently of other biomechanical exposures at work, including total sitting time, sitting with elevated upper arm, occupational physical activity and the extent of lifting and carrying in the job. Also, the level of leisure-time physical activity did not modify the association between sitting and pain, which further suggests that sitting patterns at work are independently associated with musculoskeletal health. However, because of missing values in self-reported influence and social support at work, we were not able to fully adjust for psychosocial factors, which may interact with biomechanical exposures in the development of neck–shoulder pain (Widanarko et al. 2015). Thus, this may be viewed as a limitation of the current study. Further, the data material does not allow an empirical analysis of whether the current sample was fully representative of the target population with respect to sitting exposure. However, self-reported workloads and neck–shoulder pain did not differ between those participating in the measurements and those not participating (data not shown), which suggests that our results are not afflicted by any critical selection bias.

In contrast to occupational sitting, there was no clear association between leisure-time sitting patterns and intense neck–shoulder pain. This finding corroborates a few previous studies, which did not find significant associations between total leisure-time sitting and pain (Hallman et al. 2015b; Hildebrandt et al. 2000). The reason why work and leisure would show different associations with pain is not clear. However, a viable hypothesis could be that the relationship between the temporal pattern of sitting and other exposures of relevance to neck–shoulder pain, such as sustained muscle activity following from constrained neck postures (as noted above), is more consistent, or even different, during occupational work than during leisure. Using sitting as a proxy for those exposures would then lead to more diluted and less significant results for leisure than for work. To this end, measurements of neck posture could have provided important complementary information of relevance to the interpretation of associations between sitting and pain, but it was not feasible to equip the participants with additional instrumentation.

## Conclusion

Brief and moderate periods of sitting at work showed negative (brief) and positive (moderate) associations with intense neck–shoulder pain among blue-collar workers, while prolonged periods of sitting at work did not show an association with pain. These relationships persisted after adjustment for several other established risk factors for neck–shoulder pain. Thus, our results suggest that an effect of occupational sitting on musculoskeletal health may depend on the temporal distribution of sitting. We encourage further prospective and experimental studies to disentangle the causal direction of associations between sitting and musculoskeletal pain, as well as the underlying mechanisms.
